# Photocatalytic Performance of SiO_2_/CNOs/TiO_2_ to Accelerate the Degradation of Rhodamine B under Visible Light

**DOI:** 10.3390/nano9121671

**Published:** 2019-11-22

**Authors:** Weike Zhang, Yanrong Zhang, Kai Yang, Yanqing Yang, Jia Jia, Lijun Guo

**Affiliations:** 1 School of Environmental Science and Engineering, Taiyuan University of Technology, Taiyuan 030024, China; zhangyanrong0696@link.tyut.edu.cn (Y.Z.); jiajia6328@163.com (J.J.); 15234845580@163.com (L.G.); 2 Laboratory for Earth Surface Processes, College of Urban and Environmental Sciences, Peking University, Beijing 100871, China; yangkai@pku.edu.cn

**Keywords:** titanium dioxide, carbon nano onions, silicon dioxide, rhodamine B, photocatalytic degradation

## Abstract

A silicon dioxide/carbon nano onions/titanium dioxide (SiO_2_/CNOs/TiO_2_) composite was synthesized by a simple sol-gel method and characterized by the methods of X-ray diffraction (XRD), scanning electronic microscope (SEM), X-ray photoelectron spectroscopy (XPS), Brunauer–Emmett–Teller (BET), Fourier transform infrared (FTIR), thermogravimetric analysis (TG), differential scanning calorimeter (DSC) and UV-Vis diffuse reflectance spectra (UV-Vis DRS). In this work, the photocatalytic activity of the SiO_2_/CNOs/TiO_2_ photocatalyst was assessed by testing the degradation rate of Rhodamine B (RhB) under visible light. The results indicated that the samples exhibited the best photocatalytic activity when the composite consisted of 3% CNOs and the optimum dosage of SiO_2_/CNOs/TiO_2_(3%) was 1.5 g/L as evidenced by the highest RhB degradation rate (96%). The SiO_2_/CNOs/TiO_2_ composite greatly improved the quantum efficiency of TiO_2_. This work provides a new option for the modification of subsequent nanocomposite oxide nanoparticles.

## 1. Introduction

Industrial dye wastewater is the main source of water pollution. As a triphenylmethane derivative, Rhodamine B is widely used in the manufacture of paints, acrylic fabrics and other biological products due to its bright color and good color-solidity. This dye wastewater is highly toxic to human beings (LD50: Oral-Mouse-887 mg/kg) due to its high chroma, strong toxicity and difficulty in degradation [1,2,3]. In addition, the low rate of removal during primary and secondary treatments observed in wastewater plants is due to their recalcitrant tendency toward aerobic conditions (emanating from the hard to breakdown compounds, such as aromatic structures) and it results in their easy carry-over into the aqueous ecosystem [4,5]. Recent research efforts have been centered on the development of novel strategies that can eliminate dyes more efficiently and economically. Semiconductor photocatalysis technology is one of the effective means to solve water polluted by organic contaminants [6,7,8]. As a semiconductor, titanium dioxide (TiO_2_) is featured by its chemical stability, being environmentally harmless and low cost [9,10,11,12]. However, its application in environmental pollution remediation was hampered by some internal defects of TiO_2_, such as a large band gap, higher recombination of photogenerated *e^−^*-*h^+^* pairs, small surface area and low recovery rate [13,14,15]. Therefore, the nano-TiO_2_ were modified to prepare a composite, which may have properties of a low rate of recombination of photogenerated *e*^−^-*h^+^*, high surface adsorption and a good recovery rate.

Carbon materials, such as activated carbon, carbon nanotubes, graphene, fullerene materials (C60) and graphene-based material, have been widely used in the field of photocatalysis. These carbon materials can improve photocatalytic activity by facilitating the transfer of photogenerated electrons and enhancing the adsorption performance of the catalyst due to their unique electrical properties and large specific surface area [16,17,18,19,20]. Commercial CNOs prepared by the chemical vapor deposition (CVD) method using iron-nickel as a catalyst exhibit good electrical conductivity and certain paramagnetism [21,22]. Zhang et al. synthesized Bi_2_WO_6_/MCNOs (MCNOs, magnetic carbon nano onions) via a simple hydrothermal method and reported that the degradation efficiency of RhB by the Bi_2_WO_6_/MCNOs after six cycles was 87.2% [23]. Meanwhile, our previous research found that the pure TiO_2_, CNOs/TiO_2_ (10%) composite was more effective in the separation of *e^−^*-*h^+^* and easily recovered by an external magnet (the degradation value of RhB was 78%). The reason for this is that the good electrical conductivity of nano-onion carbon was beneficial to capture photogenerated electrons and could effectively inhibit photogenerated electron-hole pair recombination. In addition, as the paramagnetism of CNOs, the catalyst powder can be recovered by an external magnet [24].

Considering that the specific surface area of CNOs/TiO_2_ (263.442 m^2^/g) is not significantly improved compared with TiO_2_ (255.948 m^2^/g), which is not conducive to the adsorption of pollution, the CNOs/TiO_2_ composite is further modified to increase its specific surface area. SiO_2_ is a typical disordered mesoporous material. Due to its large specific surface area, uniform pore size, stable chemical properties and relatively high mechanical strength, SiO_2_ has been widely used in the fields of catalysis, separation and adsorption [25,26,27]. For example, Yaparatne et al. found that P25 (P25 is a titanium dioxide with an average particle size of 25 nm composed of anatase crystals and rutile crystals) modified TiO_2_-SiO_2_ photocatalyst films which showed ~80% loss of 2-methylisoborneol (MIB) within 1 h and resulted in ~80% Geosmin (GSM) photodegradation in 1 h, while the major species hydroxyl radicals (•OH) did not change after 10 repetitions [28]. Kim et.al reported that the SBET value of the Cellulose-SiO_2_ composite aerogel (CSG, the concentration of SiO_2_ was 10 wt %) increased to 355 m^2^/g compared with the cellulose aerogel (CG, 216 m^2^/g) [29]. In addition, Cui et al. found that the surface area of 600–5% SiO_2_-TiO_2_ nanofibers (95.96 m^2^/g) was nearly 12 times that of 600–0% SiO_2_-TiO_2_ nanofibers (8.17 m^2^/g) (the Si/Ti ratio increased from 0% to 5% and treated at 600 °C) [30].

Herein, in this work, SiO_2_/CNOs/TiO_2_ photocatalyst was synthesized for the first time via a sol-gel method and was separated from aqueous media using an external magnet. In addition, SiO_2_ significantly improved the specific surface area of SiO_2_/CNOs/TiO_2_ which was 1.87-times larger than that of TiO_2_. Moreover, this study explored the photodegradation performance of the SiO_2_/CNOs/TiO_2_ composite for RhB under visible light and proposed a possible mechanism according to the characterization of the synthesized composite and free radical capture experiments.

## 2. Materials and Methods 

### 2.1. Materials

Titanium (IV) isopropoxide (C_12_H_28_O_4_Ti), ethanol (C_2_H_6_O), nitric acid (HNO_3_), tetraethyl orthosilicate (C_8_H_20_O_4_Si), rhodamine B (C_28_H_31_ClN_2_O_3_), diacetone alcohol (C_6_H_12_O_2_), 2-methoxyethanol (C_3_H_8_O_2_), sodium sulphate (Na_2_SO_4_), dimethyl carbinol (C_3_H_8_O), ethylenediaminetetraacetic acid disodium salt (C_10_H_14_N_2_Na_2_O_8_), P-19 dispersant and benzoquinone (C_6_H_4_O_2_) were purchased from Damao Chemical Reagent Factory (Tianjin, China). All the above chemical reagents were analytical grade. CNOs were purchased from Shanxi Zhongxing Environmental and Energy Technology Co. Ltd. (Shanxi, China). The Brunauer–Emmett–Teller (BET) surface area of the CNOs was 60–80 m^2^/g and D50 was 70 nm.

### 2.2. Synthesis of SiO_2_/CNOs/TiO_2_

The SiO_2_/CNOs/TiO_2_ composite was prepared by the sol-gel method. C_12_H_28_O_4_Ti and C_8_H_20_O_4_Si were used as a titanium resource and silicon resource, respectively. The CNOs were used as functional additives. The target product designed by the authors is a composite of Anatase TiO_2_ (TiO_6_ octahedral structure with a coordination number of 6), SiO_2_ crystal (SiO_4_ tetrahedral structure with a coordination number of 4) and CNOs. Therefore, the atom ratio of Ti and Si was controlled at 3:2. The products were designated as SiO_2_/CNOs/TiO_2_(X), where X was the mass ratio of CNOs to TiO_2_. The values of X in this experiment were 1%, 2%, 3%, 5% and 10% for five different SiO_2_/CNOs/TiO_2_ samples. The detailed steps of synthesis are as follows:

- CNOs/TiO_2_ solution: Firstly, 7 mL of C_12_H_28_O_4_Ti solution was added into 3 mL of C_3_H_8_O solution and stirred at room temperature. Secondly, magnetic CNOs were added to the mixed solution. The mixed solution was then transferred dropwise to a round bottom flask containing distilled water at a dropping rate of 0.4 mL/min, when the stir and condensation system were opened. Next, 1 mL of HNO_3_ solution was added to the mixed solution at a dropping rate of 0.35 mL/min at 80 °C. Finally, CNOs/TiO_2_ solution was obtained after the solution was cooled to room temperature.
- SiO_2_ solution: Firstly, 6 mL of C_8_H_20_O_4_Si, 15 mL of C_2_H_6_O, 0.35 mL of HNO_3_ and 0.4 mL of deionized water were thoroughly mixed in a three-neck round bottom flask and stirred for 30 min. Secondly, a certain amount of mixed solution (C_2_H_6_O and HNO_3_) was added to the flask and stirred at 55 ± 3 °C for 2 h. Finally, SiO_2_ solution was obtained after the solution was cooled to room temperature.
- SiO_2_/CNOs/TiO_2_ composite: Firstly, 26.5 mL of the CNOs/TiO_2_ solution prepared in Step (a) and 16 mL of the SiO_2_ solution prepared in Step (b) were mixed and stirred for 30 min. Then, 26 mL of the diluent solution 1 (C_2_H_6_O:C_6_H_12_O_2_:HNO_3_:P-19:H_2_O = 239:31:5:1:49) was added and stirred for 30 min. Next, 42 mL of the diluent solution 2 (C_2_H_6_O:C_3_H_8_O_2_:P-19:H_2_O = 260:67:1:242) was added and stirred for 1 h. Finally, the SiO_2_/CNOs/TiO_2_ composite was obtained after the solution was oven-dried at 100 °C.

### 2.3. Characterization

The morphologies and microstructures of the SiO_2_/CNOs/TiO_2_ composite were analyzed using a field-emission scanning electron microscopy (FESEM) (JSM-6700, Joel Ltd., Tokyo, Japan) with an operating voltage of 10 kV. An X-ray diffraction (XRD) analyzer (DX-2700X, Haoyuan Instrument Co., Ltd., Dandong, China) was used to characterize its crystallographic structure and crystallographic composition, with a power set at 40 kV and 30 mA, 2θ ranging from 5° to 80° at a scanning rate of 8°/min with a Cu Kα-radiation wavelength = 1.54184 Å. The functional groups of the samples were analyzed using a Nicolet iS^10^ Fourier transform-infrared spectrometer (FTIR) (Nicolet iS^10^, Thermo Fisher Scientific, Waltham, MA, USA) in the wavenumber range of 400–4000 cm^−1^. The Brunauer–Emmett–Teller (BET) surface area and pore distribution were determined using a surface area and porosity analyzer (Quadrasorb SI, Quantachrome instruments, Boynton Beach, FL, USA). The chemical states of the main elements in the photocatalysts were explored by using an X-ray photoelectron spectroscopy (XPS) (Amicus, Shimadzu, Kyoto, Japan). The thermal stability of the samples was tested on a thermogravimetric-differential scanning calorimeter (TG-DSC) (STA449 F3, NETZSCH-Gerätebau GmbH, Selb, Germany). The tests of the optical properties of the samples were carried out on an UV-Vis diffuse reflectance spectrum (UV2550, Shimadzu, Kyoto, Japan). The electrochemical properties were analyzed by using a CHI760E electrochemical workstation which was based on a three-electrode system with a working electrode (ITO slide), an auxiliary electrode (Pt) and a reference electrode (standard calomel electrode (SCE)), while 0.5 mol/L Na_2_SO_4_ solution was used as the electrolyte.

### 2.4. Photocatalytic Degradation Experiments

The light source was a 300 W Xenon (Xe)-lamp with a cutoff filter (λ ≥ 420 nm) and a light-proof box on the outside. Rhodamine B (RhB) was selected to evaluate the photocatalytic activity of the catalytic material. The specific steps were as follows. Firstly, a given amount of the SiO_2_/CNOs/TiO_2_ sample was added to 100 mL of 10 mg/L RhB solution, followed by 10 min ultrasonic dispersion. Then, the mixture was stirred in the dark for 30 min, in which the adsorption-desorption equilibrium was reached between the catalysts and the reactants. Finally, the mixture was stirred using a magnetic stirrer with the Xe-lamp irradiating (120.5–150.0 mW/cm^2^ of the photon flux density). Further, 6 mL of the mixture was taken at an interval of 20 min and centrifuged, followed by measuring the absorbance of the supernatant with an ultraviolet spectrophotometer (UV-2102PC, Unico, Princeton, NJ, USA). 

Photocatalytic degradation efficiency was calculated by the following equation:

(1) $$D\%=\left[\frac{C_{0}-C_{t}}{C_{0}}\right]\times 100\%=\left[\frac{A_{0}-A_{t}}{A_{0}}\right]\times 100\%$$

where *C*_0_ and *C_t_* are the initial concentration (mg/L) and *t*-time equilibrium concentration (mg/L)*. A_0_* and *A_t_* are the absorbance of original and time *t*, respectively.

In addition, the total organic carbon (*TOC*) of the solution was tested using a Total Organic Carbon Analyzer (TOC) (TOC-V CPH, Shimadzu, Kyoto, Japan). The mineralization ratio was determined by the following equation [31]:

(2) $$M\%=\left[\frac{TOC_{0}-TOC_{t}}{TOC_{0}}\right]\times 100\%$$

where *TOC_0_* and *TOC_t_* are the initial concentration (mg/L) and *t*-time equilibrium concentration (mg/L), respectively.

## 3. Results and Discussion

### 3.1. Characterization of TiO_2,_ SiO_2,_ CNOs and SiO_2_/CNOs/TiO_2_(3%)

**Figure 1:**
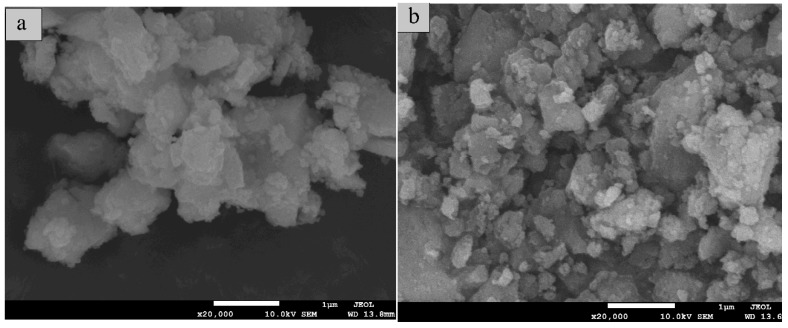
Scanning electronic microscope (SEM) images of (**a**) TiO_2_ and (**b**) silicon dioxide/carbon nano onions/titanium dioxide (SiO_2_/CNOs/TiO_2_(3%)).

The morphologies and sizes of the as-prepared samples were observed by scanning electronic microscope (SEM) images. The TiO_2_ sample was composed of particles with heavy agglomerates as its sizes were 0.5–1 um (Figure 1a). In contrast, the particles of SiO_2_/CNOs/TiO_2_ was presented with slight agglomeration as its sizes in 0.1–0.3 um (Figure 1b), indicating the dispersion of SiO_2_/CNOs/TiO_2_ improved significantly than pure TiO_2_. The high dispersion of the as-prepared composite may not only improve its adsorption capacity, but also expose more active sites. 

**Figure 2:**
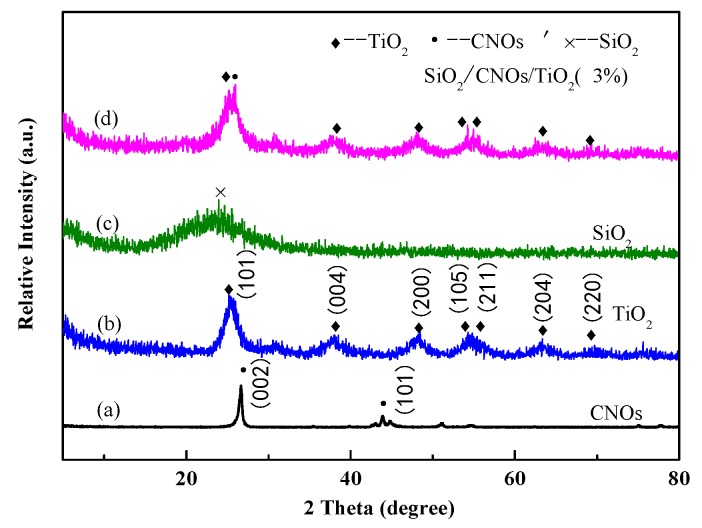
The X-ray diffraction (XRD) patterns of (**a**) CNOs, (**b**) TiO_2_, (**c**) SiO_2_ and (**d**) SiO_2_/CNOs/TiO_2_(3%).

The XRD patterns of CNOs, TiO_2_, SiO_2_ and SiO_2_/CNOs/TiO_2_(3%) offered some information about the phase compositions and crystal properties in Figure 2. In Figure 2a, there was an obvious diffraction peak of CNOs at 26.7°, corresponding to the (002) crystal plane of the graphitized cubic crystal [21]. In Figure 2b, the diffraction peaks (25.2° (101), 37.8° (004), 48.1° (200), 53.9° (105), 55.1° (211), 62.7° (204), and 70.3° (220)) were well assigned to the anatase diffraction peaks (PDF#21-1272), indicating that the synthesized TiO_2_ was anatase [32]. In Figure 2c, there was a broad peak at 23° of SiO_2_, a characteristic peak of amorphous SiO_2_, indicating that SiO_2_ existed in an amorphous state [33]. In Figure 2d, the diffraction peaks of SiO_2_/CNOs/TiO_2_(3%) were similar to those of pure TiO_2_, indicating that the form of TiO_2_ existed in the composite was anatase.

The characteristic diffraction peak at 26.7° belonging to the CNOs (002) crystal plane was also observed, indicating that CNOs effectively combined with TiO_2_. However, there were no obvious diffraction peaks of SiO_2_ probably due to the amorphous state of SiO_2_.

The diffraction peaks at the (104) and (200) crystal planes in TiO_2_ were selected to calculate the grain size using the Scherrer formula. These peaks could avoid interference of diffraction peaks between the (101) crystal plane of TiO_2_ and the (002) plane of CNOs. As shown in Table 1, the grain size of SiO_2_/CNOs/TiO_2_(3%) was smaller than that of TiO_2_ and CNOs/TiO_2_. This finding was consistent with the above SEM images, indicating that the addition of SiO_2_ improved the dispersity of the sample.

**Figure 3:**
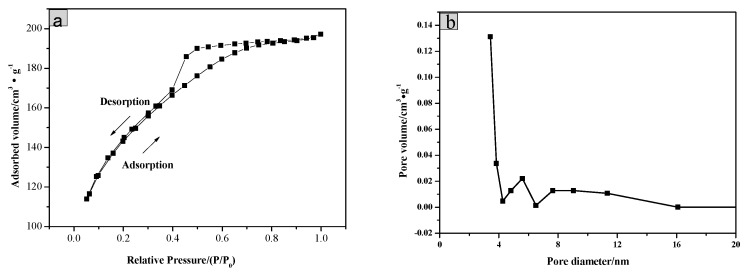
(**a**) Nitrogen adsorption/desorption isotherms and (**b**) pore size distribution of SiO_2_/CNOs/TiO_2_(3%).

The BET surface area of the as-prepared SiO_2_/CNOs/TiO_2_(3%) was evaluated by the nitrogen adsorption and desorption measurement (Figure 3). The sample showed a type IV isotherm with a H_2_ hysteresis loop in Figure 3a, belonging to the porous structure type predominated by mesoporous. Meanwhile, its average pore size was 3.54 nm as presented in the pore size distribution of the SiO_2_/CNOs/TiO_2_(3%) composite (Figure 3b), confirming that the sample was a mesoporous structure.

The specific surface area, average pore size and pore volume of the samples are shown in Table 1. Due to the addition of SiO_2_ and CNOs, the specific surface area of SiO_2_/CNOs/TiO_2_(3%) was 1.87-times larger than that of TiO_2_. The improvement in the specific surface area may be beneficial to pollutant adsorption. 

**Figure 4:**
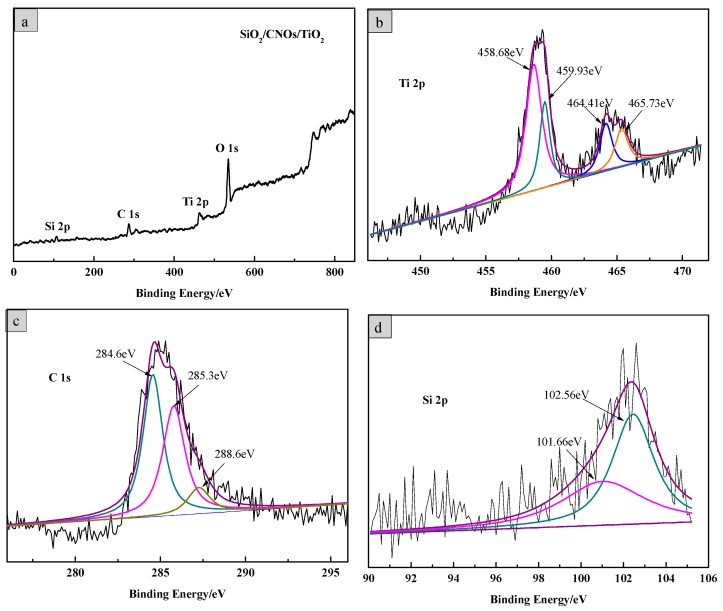
(**a**) X-ray photoelectron spectroscopy (XPS) full spectrum of the SiO_2_/CNOs/TiO_2_(3%) sample and high-resolution spectra of (**b**) Ti, (**c**) C and (**d**) Si.

The element composition and valence bond structure of the SiO_2_/CNOs/TiO_2_(3%) composite were characterized by XPS analysis. Figure 4 shows the X-ray photoelectron full spectrum of SiO_2_/CNOs/TiO_2_(3%) and high-resolution spectra of Ti 2p, C 1s and Si 2p, respectively. For the SiO_2_/CNOs/TiO_2_(3%) composite, there were four predominant binding energy peaks at 459 eV, 285 eV, 102 eV and 530 eV, corresponding to the Ti 2p, C 1s, Si 2p and O1s, respectively. In the spectrum of the Ti element (Figure 4b), two characteristic peaks observed at approximately 459.7 eV and 465.1 eV corresponded to Ti^4+^ 2p_1/2_ and Ti^4+^ 2p_3/2_ in anatase TiO_2_, indicating that the Ti element existed as Ti^4+^ in the sample [34]. Meanwhile, the peaks at 460.9 eV and 466.7 eV corresponded to Ti 2p in Ti-O-Si. In the high-resolution spectrum of the C element (Figure 4c), the peaks at the binding energies of 284.6 eV, 283.3 eV and 288.6 eV corresponded to the contamination of the instrument itself, sp^2^ hybridization in CNOs or C of Ti–O–C bonds and the C=O in CNOs, respectively [35,36]. There were two peaks at 102.56 eV and 101.66 eV in the high-resolution spectrum of the Si element (Figure 4d), which belonged to the binding energy Si 2p in Si-O-Si and Ti-O-Si, respectively [37]. The XPS results are consistent with the results of the XRD analysis, confirming that the SiO_2_/CNOs/TiO_2_(3%) composite was successfully synthetized.

**Figure 5:**
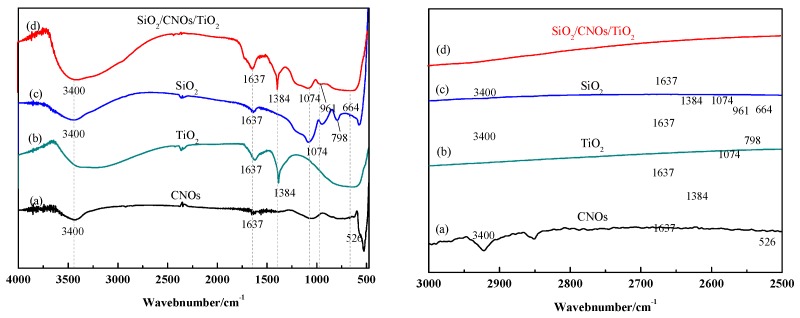
Fourier transform-infrared spectrometer (FTIR) spectra of (**a**) CNOs, (**b**) TiO_2_, (**c**) SiO_2_ and (**d**) SiO_2_/CNOs/TiO_2_(3%) and partial enlargement in 3000–2500 cm^−1^.

Figure 5 shows the FTIR spectra of TiO_2_, CNOs, SiO_2_ and SiO_2_/CNOs/TiO_2_(3%). The absorption peaks near 3400 cm^−1^ and 1637 cm^−1^ of all the samples may be caused by water molecules in the samples or the stretching vibration of the hydroxyl functional groups [38]. For CNOs (Figure 5a): The characteristic absorption peaks at 2900 cm^−1^ and 2820 cm^−1^ corresponded to the stretching vibration of the methyl and methylene groups; the peak at 1064 cm^−1^ was caused by C–O bond stretching vibration; the peak at 526 cm^−1^ corresponded to the C60 energy band [39,40]. For TiO_2_ (Figure 5b), the symmetry stretching vibration peak of the Ti–O–Ti bond was located at 604 cm^−1^ [41], and the bending vibration peak of NO_3_^−^ was located at 1384 cm^−^^1^ due to the addition of HNO_3_ in the material synthesis. In the infrared spectrum of SiO_2_ (Figure 5c), the characteristic peaks at 956 cm^−1^ and 1074 cm^−1^ were the symmetric stretching vibration of Si–O–Si and the antisymmetric stretching vibration of Si–O–Si, respectively [42]. For the SiO_2_/CNOs/TiO_2_ (3%) composite (Figure 5d), the characteristic peaks at 1384 cm^−1^, 664 cm^−1^ and 961 cm^−1^ corresponded to the bending vibration of the C–C bond, the symmetric stretching vibration of Ti–O–Ti bond and the stretching vibration of Si–O–Si, respectively. Meanwhile, the characteristic peak of CNOs at 1064 cm^−1^ was weak due to its overlap with the peak of the antisymmetric stretching vibration of Si-O-Si (1074 cm^−1^). The results indicated that the composite was successfully synthesized by TiO_2_, SiO_2_ and CNOs [38,39,40,41,42].

**Figure 6:**
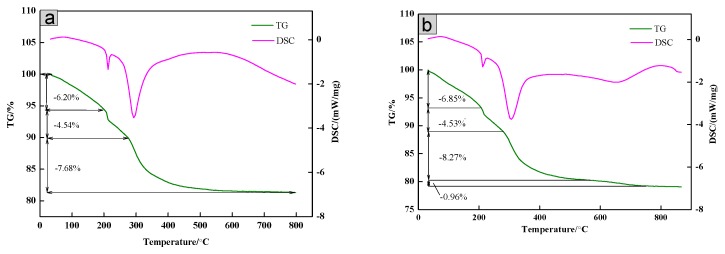
Thermogravimetric-differential scanning calorimeter (TG-DSC) curves of (**a**) TiO_2_ and (**b**) the SiO_2_/CNOs/TiO_2_(3%) composite.

The thermal stability of the SiO_2_/CNOs/TiO_2_(3%) composite was explored by TG-DSC. In Figure 6a, the thermogravimetric analysis (TG) curve of the TiO_2_ sample consisted of four parts. Part 1: below 200 °C. The mass loss was 6.23 wt %, derived from moisture evaporation from the TiO_2_ surface. Meanwhile, the value of the DSC curve was greater than zero, indicating that the process was an endothermic reaction. Part 2: 200–300 °C. The weight loss was 4.51 wt %, caused by the dihydroxylation process inside the material. During the process, the hydroxyl group combined oxygen to form water. Part 3: 300–600 °C. The weight loss was 7.68%, derived from the oxidative decomposition of organic matter in the sample. Part 4: above 600 °C. The main component in the remaining sample was pure TiO_2_ without mass lost [40]. The TG curve of SiO_2_/CNOs/TiO_2_(3%) was made up of five parts (Figure 6b). The mass loss of the composite before 600 °C was similar to that of the TiO_2_ sample, of which the weight loss of evaporation, dihydroxylation and oxidative decomposition of the organic matter were 6.85%, 4.53% and 8.27%, respectively. Between 550 °C and 780 °C, the mass loss of the sample was 0.96%, caused by the thermal decomposition of CNOs in the sample. Above 780 °C, the weight remained stable. The residual was dominated by SiO_2_ and TiO_2_ [43]. These results indicated that the thermal stability of the SiO_2_/CNOs/TiO_2_(3%) composites was quite good.

**Figure 7:**
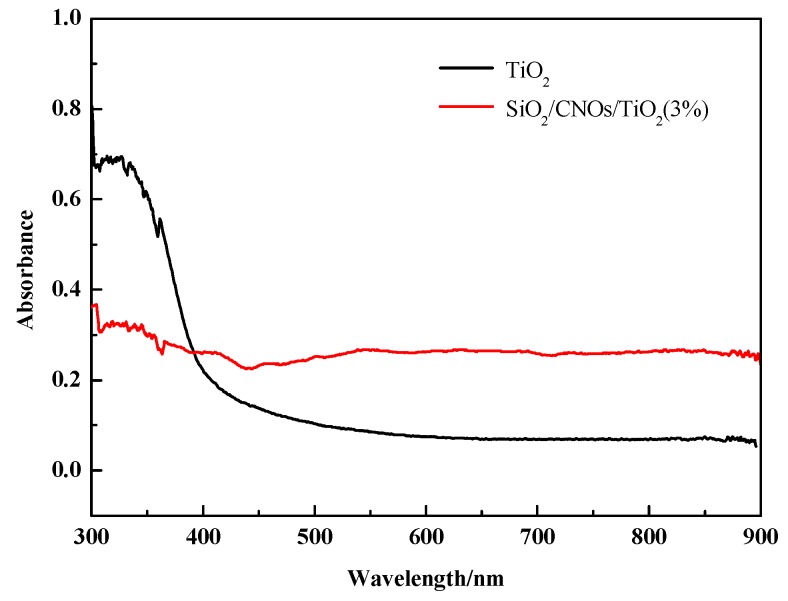
UV-Vis diffuse reflectance spectra of TiO_2_ and SiO_2_/CNOs/TiO_2_(3%).

The band gap energies were estimated using the Tauc formula (*αhν*)^1/2^∝ (*hυ*–*Eg*), where α is the absorption coefficient, ν is the frequency of the light, h is Planck’s constant and Eg is the band gap [44,45]. In Figure 7, the absorbance of SiO_2_/CNOs/TiO_2_(3%) in the visible region was stronger than TiO_2_, in which the band gap energies were found to be 2.90 eV and 2.22 eV for TiO_2_ and SiO_2_/CNOs/TiO_2_(3%).

In addition, the conduction band (CB) and valence band (VB) of TiO_2_ are calculated by following equations:

*E^0^_CB_* = *χ* − *E^C^* − 0.5*Eg* (3)

*E_VB_* = *E_CB_* − *Eg* (4)

where χ is the absolute electronegativity of the semiconductor (χ is 5.81 eV for TiO_2_ ), *E^C^* is the energy of free electrons on the hydrogen scale (~4.5 eV) and Eg is the band gap energy of the semiconductor. After calculations, the VB and CB of TiO_2_ are −3.04 eV and −0.14 eV, respectively. There may be two possible reasons. On the one hand, the addition of CNOs improved the visible light absorption capacity of TiO_2_ due to its high electrical conductivity [21,22]. On the other hand, the addition of SiO_2_ enhanced the dispersibility of the material caused by its large specific surface area and therefore increased the contact rate of the material with photons under visible light [29,30].

### 3.2. Degradation of RhB under Visible Light Irradiation

**Figure 8:**
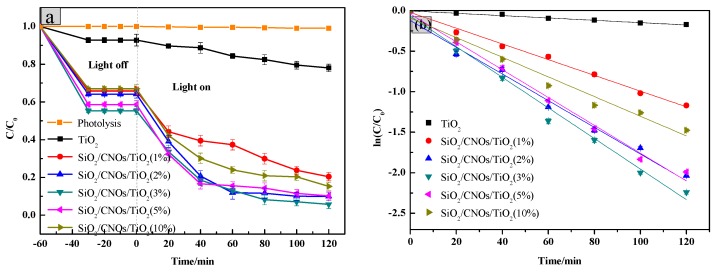
(**a**) Photocatalytic degradation of different CNOs composite ratio and (**b**) linear transform of ln(C/C_0_) = k·t of the kinetic curve of Rhodamine B degradation by the TiO_2_/CNOs/ SiO_2_ catalysts.

Photocatalytic performance was evaluated by measuring the degradation efficiency of RhB (100 mL of 10 mg/L RhB, dosage was 2.0 g/L). Photocatalytic degradation efficiencies of RhB by different ratio CNOs to the SiO_2_/CNOs/TiO_2_ composites is presented in Figure 8. 

In the dark reaction, all samples reached the adsorption-desorption equilibrium in 30 min and the process could adsorb approximately 35–45% of RhB corresponding to the adsorbed quantities which were 1.634–2.274 mg/L as seen in Figure 8a. This phenomenon may be attributed to the large specific surface area of SiO_2_ which was beneficial to the adsorption of pollutants. In the light reaction, compared with the absence of the photocatalyst, the degradation efficiency of RhB significantly improved after the addition of the prepared materials. The low CNOs (<3%) may have a little high recombination of photogenerated *e^−^*-*h^+^* as the e^−^ could not transfer to CNOs immediately. On the other hand, the high-level CNOs (>3%) may lead the formation of passive layers that lowered the photoadsorption efficiency of SiO_2_/CNOs/TiO_2_ and reduced the specific surface area. Meanwhile, the degradation efficiency of SiO_2_/CNOs/TiO_2_(3%) reached the highest value of 94% (Figure 8a), so 3% was selected as the optimal content of CNOs in this work. 

To further explore the catalytic reaction, the degradation kinetics of the catalyst with the integrated rate law ln(C/C_0_) = k∙t (Figure 8b) was investigated [46]. As shown in Figure 8b, the rate constants k were −0.00148, −0.00956, −0.01714, −0.01815, −0.01638 and −0.01213 min^−1^ for TiO_2_, SiO_2_/CNOs/TiO_2_(1%), SiO_2_/CNOs/TiO_2_(2%), SiO_2_/CNOs/TiO_2_(3%), SiO_2_/CNOs/TiO_2_(5%) and SiO_2_/CNOs/TiO_2_(10%), respectively. The absolute value of k of SiO_2_/CNOs/TiO_2_(3%) was largest, which was consistent with the SiO_2_/CNOs/TiO_2_(3%) composites which exhibited optimal photoactivity.

**Figure 9:**
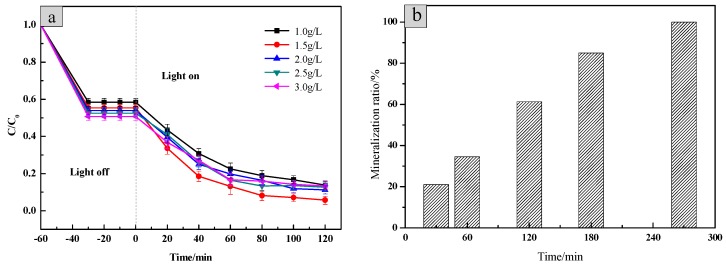
(**a**) The effect of the dosage of SiO_2_/CNOs/TiO_2_(3%) on photodegradation efficiency and (**b**) the RhB’s mineralization ratio of 1.5 g/L SiO_2_/CNOs/TiO_2_(3%) during photocatalytic processes.

In order to explore the effect of the sample dosage on photodegradation efficiency, 1.0 g/L, 1.5 g/L, 2.0 g/L, 2.5 g/L and 3.0 g/L of the SiO_2_/CNOs/TiO_2_(3%) composite were added to 100 mL of 10 mg/L RhB solution, respectively (Figure 9).

At the beginning, the photodegradation efficiency of RhB increased with the increase of the amount of SiO_2_/CNOs/TiO_2_(3%). Then, it decreased as the amount of the sample increased continuously, caused by high turbidity in the reaction system and aggregation of the catalyst [23]. The optimum additional amount of SiO_2_/CNOs/TiO_2_(3%) was 1.5 g/L as the value of the photodegradation efficiency for RhB reached 96.25% after 120 min under visible light irradiation. To further explore the kinetics of RhB photodegradation, the mineralization ratio by TOC was measured [31]. As shown in Figure 9b, after 120 min and 270 min of irradiation, the mineralization ratio of RhB reached 61.31% and 100%, respectively.

**Figure 10:**
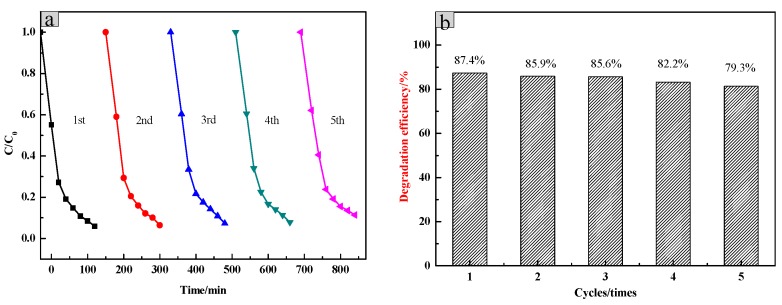
(**a**) The five-run test of photocatalytic activity by using the recovered SiO_2_/CNOs/TiO_2_(3%) and (**b**) its degradation efficiency.

The stability of the SiO_2_/CNOs/TiO_2_(3%) was determined by the efficiencies of the repeated degradation of RhB (100 mL of 10 mg/L RhB, dosage was 1.5 g/L). The catalyst material was recovered by applying an external magnet due to the paramagnetism magnetism of CNOs, which was washed several times with distilled water and subsequently dried at 80 °C for 12 h. The degradation efficiency of the recovered catalyst presented in Figure 10 shows that the degradation efficiency of RhB reduced from 96.25% in the initial test to 79.3% after 5 cycles of testing. This may be due to a decrease in the surface-active sites of the catalyst or a mass loss of the catalyst during the recovery process.

**Figure 11:**
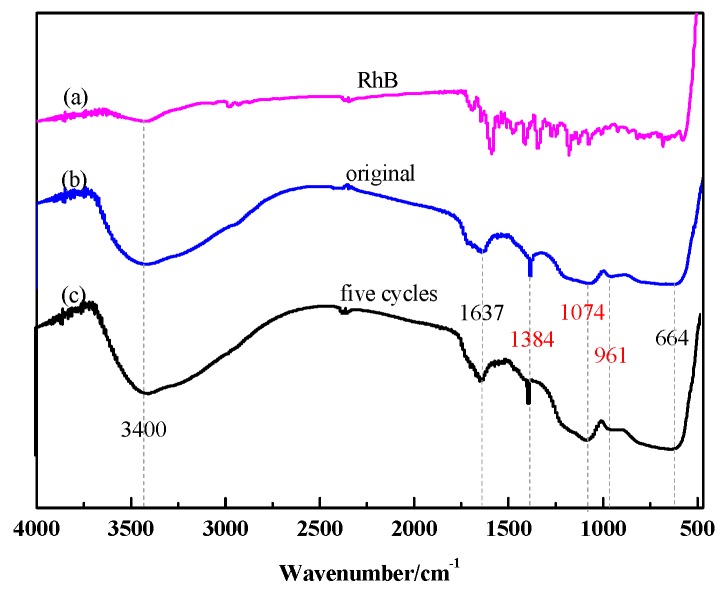
(**a**) The FTIR spectra of RhB, (**b**) original SiO_2_/CNOs/TiO_2_(3%) and (**c**) five cycles of the SiO_2_/CNOs/TiO_2_(3%).

Furthermore, in order to investigate the cycle stability of the catalyst, RhB, the newly prepared SiO_2_/CNOs/TiO_2_(3%) composite and the five cycles composite were characterized by FTIR, respectively (Figure 11). The intensity and position of the characteristic absorption peak of the two catalysts hardly changed. No other new absorption peak was found in the five cycles of the catalyst indicating that the adsorbed RhB on the photocatalytic material was completely degraded. The above results showed that the SiO_2_/CNOs/TiO_2_(3%) composite had good stability.

### 3.3. Photodegradation Mechanism of the SiO_2_/CNOs/TiO_2_(3%) Composite

**Figure 12:**
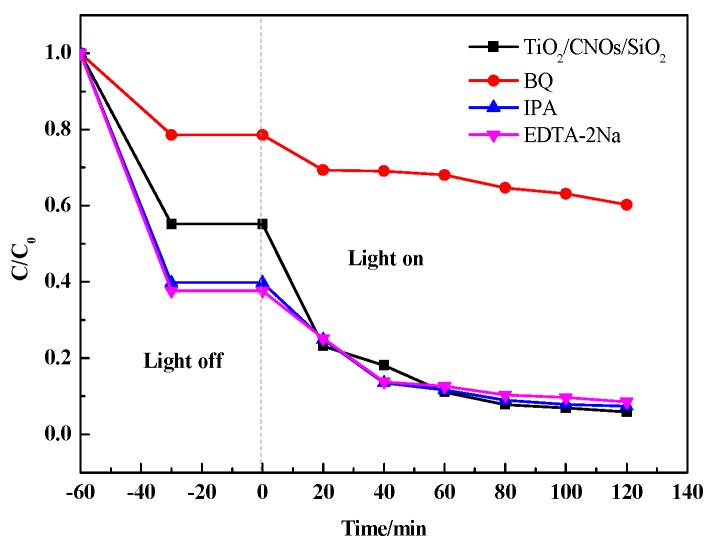
Reactive species trapping experiments of RhB with the sample SiO_2_/CNOs/TiO_2_(3%).

In order to investigate the role of the different active species in the catalytic system which was generated under visible light irradiation, EDTA-2Na (0.02 g/L) was used to capture *h^+^*. Isopropanol (IPA) (0.02 g/L) was added to capture •OH and benzoquinone (BQ) (0.01 g/L) was adopted to capture •O_2_^−^, respectively [47]. In Figure 12, compared with the original degradation, the degradation efficiency of RhB did not change significantly after adding IPA and EDTA-2Na. However, the efficiency of the photocatalytic degradation reduced from 94% to 60% when BQ was added, indicating that the •O_2_^−^ was the main active species in the photocatalytic degradation process.

**Figure 13:**
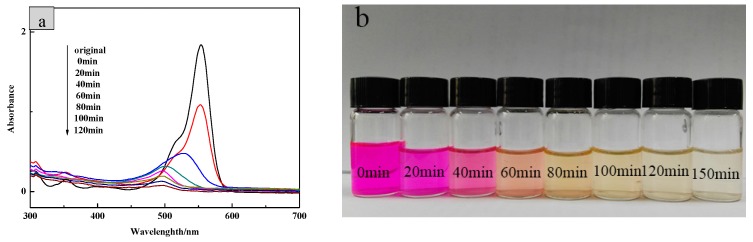
(**a**) UV-vis scanning of different degradation time of RhB solution and (**b**) the color change of RhB during photodegradation.

Then, the UV-visible absorption spectrum of RhB solutions with different degradation time was measured. In Figure 13, the reaction time was extended until the RhB solution degraded and became colorless with the optimum dosage of 1.5 g/L. It can be seen from the figure that the intensity of the largest characteristic absorption peak at 554 nm was significantly weakened and its position was blue-shifted within the progress. The entire degradation process took 150 min, in which the color of the RhB solution changed from pink to light yellow, and finally to colorless.

**Figure 14:**
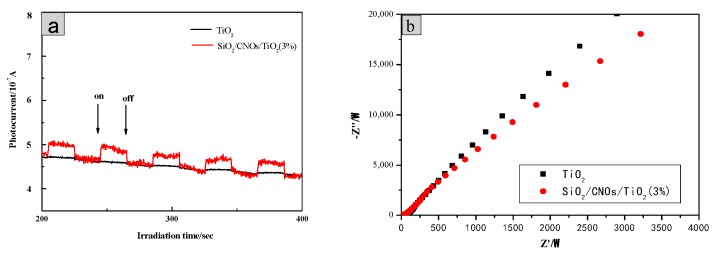
(**a**) Time-based photocurrent responses of samples and (**b**) Nyquist plots.

In addition, the excitation and migration of photogenerated carriers during photocatalysis was explored by photochemical tests. In Figure 14a, for the SiO_2_/CNOs/TiO_2_(3%) composite, the value of photocurrent response did not change significantly after several tests, indicating that the photocurrent response of the material was stable. Meanwhile, the prepared sample showed a higher photocurrent intensity than TiO_2_ when the light source was turned on, indicating that the composite enhanced the separation efficiency of electrons and holes [48].

The electron mobility of photocatalytic materials was further studied according to an electrochemical impedance spectroscopy (EIS) test (Figure 14b. The charge transport resistance of the SiO_2_/CNOs/TiO_2_(3%) material was smaller than that of TiO_2_ as the radius of SiO_2_/CNOs/TiO_2_(3%) was smaller, increasing the electron migration rate and reducing the recombination probability of photogenerated *e*^−^-*h^+^* pairs.

**Figure 15:**
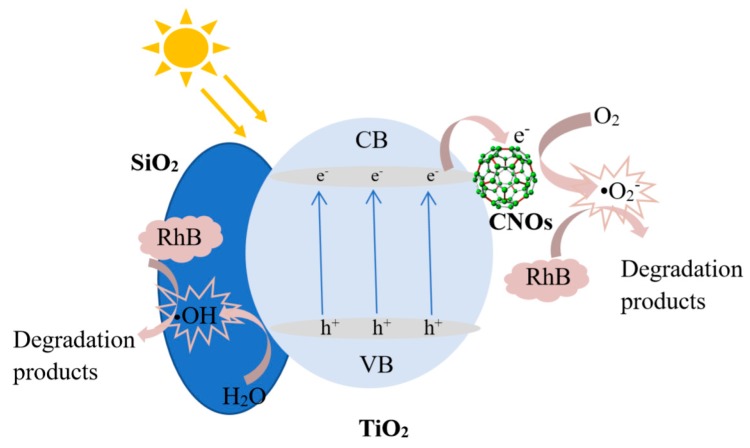
The mechanism diagram of SiO_2_/CNOs/TiO_2_(3%) in the degradation of dyes in visible light.

Based on the above results, the photocatalytic degradation mechanism of the SiO_2_/CNOs/TiO_2_(3%) composite may be elucidated as follows. First, the BET surface area of the SiO_2_/CNOs/TiO_2_(3%) sample was 1.94-times larger than that of the pure TiO_2_ (Table 1), benefitting the adsorption of RhB in a dark reaction and the contact between catalysts and contaminants. Then, after N-deethylation of RhB, the opening ring process began with the formation of oxides and small molecule compounds, which were further mineralized into Cl^−^, CO_2_, NO_3_^−^, NH_4_^+^ and H_2_O [49,50]. Figure 15 shows the reactions that may occur during photocatalysis. For the SiO_2_/CNOs/TiO_2_(3%) composite under light irradiation, the electrons (*e*) are excited and transfer from the valence band (VB) to the conduction band (CB), forming *e*^−^-*h^+^* pairs that travel to the catalyst surface. The band gap energy was 2.22 eV of SiO_2_/CNOs/TiO_2_(3%) (Figure 7). As CNOs have good conductivity, photogenerated electrons of the prepared composite transferred to CNOs and reacted with O_2_ in the pollutants to form superoxide anion •O_2_^−^(main active species), resulting in the separation of photogenerated electrons from holes [51]. Moreover, due to the high surface content of the Ti–O–Si species (formed between TiO_2_ and SiO_2_), which improved dispersion effectively, the photocatalytic degradation of RhB was significantly enhanced [52].

The reactions that occurred in this process were as follows.

SiO_2_/CNOs/TiO_2_(3%) + *hν* →SiO_2_/CNOs(*e^−^*)/TiO_2_ (*h^+^*) (5)

TiO_2_ (*h^+^*) + H_2_O → •OH + H^+^ +TiO_2_ (6)

OH- + TiO_2_ (*h^+^)* → •OH + TiO_2_ (7)

O_2_ + CNOs (*e^−^*) →•O_2_^−^ (8)

RhB + •O_2_^−^ → CO_2_ + H_2_O+NO_3_^−^+NH_4_^+^ +Cl^−^ (9)

## 4. Conclusions

The SiO_2_/CNOs/TiO_2_(3%) composite was successfully prepared by a sol-gel method with a large surface area of 497 m^2^/g. The good electrical conductivity of CNOs and the strong adsorption properties of SiO_2_ benefited the migration of electrons and separation efficiency of the photo-generated *e^−^*-*h^+^* pairs. When the compounding amount of CNOs was 3% and the dosage was 1.5 g/L, the composite showed the highest photocatalytic activity, where the degradation rate of RhB reached 96.25%. In addition, due to the paramagnetism of the CNOs, the powders can be easily recovered from the aqueous solution using an external magnet. Furthermore, the possible photocatalytic mechanism of the SiO_2_/CNOs/TiO_2_(3%) composite was proposed based on the all experiments. This work may provide a new insight to improve the performance of photocatalysts.

## Figures and Tables

**Table 1:** Grain size, surface area, average pore size and pore volume of TiO_2_, CNOs/TiO_2_(10%) and SiO_2_/CNOs/TiO_2_(3%).

Sample	Grain Size (nm)	Specific Surface Area(m^2^/g)	Average Pore Size (nm)	Pore Volume (cm^3^/g)
TiO_2_	26.53	255.948	2.348	0.150
CNOs/TiO_2_(10%)	23.54	263.442	2.367	0.156
SiO_2_/CNOs/TiO_2_(3%)	22.55	479.243	3.54	0.305
